# Anthropometric Measurements, Metabolic Profile and Physical Fitness in a Sample of Spanish Women with Type 2 Diabetes

**DOI:** 10.3390/ijerph182211955

**Published:** 2021-11-14

**Authors:** María Orosia Lucha-López, Concepción Vidal-Peracho, César Hidalgo-García, Jacobo Rodríguez-Sanz, Héctor Tricás-Vidal, Mar Hernández-Secorún, Sofía Monti-Ballano, José Miguel Tricás-Moreno, Ana Carmen Lucha-López

**Affiliations:** 1 Physiotherapy Research Unit, Faculty of Health Sciences, University of Zaragoza, C/Domingo Miral s/n, 50009 Zaragoza, Spain; cvidal@unizar.es (C.V.-P.); marhsecorun@unizar.es (M.H.-S.); 681265@unizar.es (S.M.-B.); jmtricas@unizar.es (J.M.T.-M.); analucha@unizar.es (A.C.L.-L.); 2 Specialty Medical Centre Grande Covián, SALUD, Avda. Alcalde Caballero, 196, 50014 Zaragoza, Spain; 3 Faculty of Medicine and Health Sciences, Universitat Internacional de Catalunya, Sant Cugat del Vallès, 08195 Barcelona, Spain; jrodriguezs@uic.es; 4 Physiotherapy Research Unit, University of Zaragoza, C/Domingo Miral s/n, 50009 Zaragoza, Spain; hjtricas@gmail.com

**Keywords:** type 2 diabetes, exercise training, cardiovascular risk, endocrinology

## Abstract

Background: Exercise training has proven to be effective for treatment of metabolic diseases, such as type 2 diabetes mellitus. The aims of this study were to compare anthropometric measurements, metabolic profile and physical fitness between active and sedentary women with type 2 diabetes, and to analyse relationships between anthropometry and metabolic profile and components of physical fitness (balance, flexibility, strength and endurance). Methods: Cross-sectional research on 28 women with type 2 diabetes. Amount of daily physical activity, BMI, waist circumference, HbA1c, fibrinogen, hs-CRP, tiptoe dynamic balance, static balance, finger floor distance, abdominal, upper and lower limb strength and walking cardiovascular endurance were recorded. Results: Age: 58.5 ± 7.8. Overall, 16 subjects were physically active and 12 were sedentary. Active subjects had lower BMI (*p* = 0.033) and better cardiovascular endurance (*p* = 0.025). BMI and waist circumference were not influenced by any physical fitness component. HbA1c, fibrinogen and hs-CRP were related with worse dynamic balance (*p* = 0.036, 0.006 and 0.031, respectively). Conclusions: Active women had lower BMI and showed a better performance in cardiovascular endurance. Tiptoe dynamic balance impairments were related to worse glycaemic control, hypercoagulation and inflammatory state.

## 1. Introduction

Exercise training has proven to be effective for prevention and treatment of several obesity-related metabolic diseases, such as type 2 diabetes mellitus [1].

Prospective observational studies and clinical trials have found an inverse relationship between physical activity and risk of diabetes and cardiovascular disease, which persists even after having eliminated body mass index (BMI), which suggests that the metabolic and cardiovascular benefits derived from physical activity are not only explained by weight regulation [2,3]. 

Physical training significantly improves glycaemic control and reduces visceral adipose tissue and plasma triglycerides in people with type 2 diabetes mellitus, even without weight loss [4]. Observational data or data from clinical trials suggest that regular physical activity, alone or combined with dietary therapy, improves insulin sensitivity, glycaemic control and metabolic profile between diabetic and nondiabetic populations [5]. The Insulin Resistance Atherosclerosis Study shows significant relationships between moderate or vigorous physical activity and insulin sensitivity among middle-aged men and women [6]. Increases in moderate or vigorous physical activity of 200 kilocalories/day were associated with augmentations in insulin sensitivity of 2.9 and 2.6, respectively. After adjusting for BMI and for waist–hip circumference, the increments decreased (1.7 and 1.9, respectively), although they were still significant. In the British Regional Heart Study, both moderate and vigorous activity had an inverse relationship with the level of fasting insulinemia. In addition, reductions in body and abdominal fat, regardless of weight loss, were associated with improvements in insulin sensitivity [7].

Exercise alone, in the absence of changes in body composition, is capable of improving glucose homeostasis [3]. During exercise, contractions increase blood glucose uptake to supplement intramuscular glycogenolysis [8]. This effect persists for several hours [9], even when insulin-mediated uptake is impaired by type 2 diabetes mellitus [10].

This observation does not negate the strong deleterious effect of overweight, given that there is considerable evidence associating dyslipidaemia and insulin resistance with increases in body fat, and in particular with visceral adiposity [11]. During weight gain, pathological expansion of adipose tissue leads to inflamed, fibrotic, and dysfunctional tissue that stimulates insulin resistance [12]. 

Diabetes is linked to augmented circulating inflammatory biomarkers including C-reactive protein and fibrinogen. Hyperglycaemia induces oxidative stress and the generation of highly reactive products that induce structural modifications and functional impairments of various proteins, including fibrinogen [13]. Previous studies have reported that there is a relationship between higher levels of circulating C-reactive protein, related to adiposity and diabetes [14]. Moderate-intensity exercise has been shown to ameliorate serum levels of high-sensitivity C-reactive protein and fibrinogen in patients with type 2 diabetes mellitus [15]. 

Recommendations to treat or prevent obesity and type 2 diabetes mellitus in humans via physical activity have focused on aerobic endurance training. The American Diabetes Association (ADA), in the Standards of Medical Care in Diabetes 2014, stated that adults with diabetes should be advised to perform at least 150 min/week of moderate-intensity aerobic physical activity (50–70% of maximum heart rate), spread over at least 3 days/week with no more than 2 consecutive days without exercise [16]. Aerobic physical activity has been shown to favour weight loss [17] and improvements of insulin sensitivity and glucose metabolism [18] in obesity and type 2 diabetes mellitus.

Briefly, more physical activity is associated with lower type 2 diabetes mellitus, obesity and cardiovascular risk. However, there is little evidence available describing the physical fitness condition of different subgroups of diabetic patients. The aims of this study were to compare anthropometric measurements, metabolic profiles and physical fitness between active and sedentary women with type 2 diabetes, and to analyse relationships between anthropometry and metabolic profile and components of physical fitness (balance, flexibility, strength and endurance).

## 2. Materials and Methods

A nonexperimental cross-sectional study was performed, with ex post facto comparative and regression analysis, in a sample of 28 white women with type 2 diabetes who attended specialized Endocrinology and Nutrition outpatient clinic at the Hospital Royo Villanova (Grande Covian Specialty Medical Centre) in Zaragoza, Spain.

Inclusion criteria were women with diagnosis of type 2 diabetes, according to criteria of the ADA [19], and being older than 45 years [20]. All patients attending the consultation who met the inclusion criteria were asked to participate. They voluntarily accepted participation after being informed about the study, and we constructed a convenient, consecutive, nonprobabilistic sample method. All participants signed an informed consent declaration, and they were informed that they could leave the study at any time and for any reason. The Department of Physical Therapy and Nursing in University of Zaragoza authorized the study, which complied with the ethical requirements of the Declaration of Helsinki [21]. 

### 2.1. Measures

#### 2.1.1. Amount of Daily Physical Activity Performed by the Subjects

Seven-Day Physical Activity Recall is an instrument that reflects the physical activity that takes place for seven days a week [22]. Time and intensity (moderate, vigorous or very vigorous) are reflected, describing the activity due to work or occupation and leisure activities.

This variable allowed classification of the subjects into physically active (they did some physical activity of any intensity level) or sedentary.

#### 2.1.2. Anthropometric Measurements

Body weight and height were obtained in subjects wearing light clothing and no shoes. Data were obtained according to the protocol of the International Society for the Advancement of Kinanthropometry [23]. Height was measured with a wall stadiometer (Seca-Health Line: removable stadiometer, scope 30–220 cm), weight with an electronic scale (Biological Company: TANITA TBF 300) and waist circumference with inextensible tape (TECSYMP Instruments: plastic tape, measure 0–2 m). BMI was calculated as weight in kilograms divided by the square of the height in metres. 

#### 2.1.3. Laboratory Assessments

Blood samples were collected after a fasting period of not less than 12 h by venepuncture. Samples were then transferred to the Grande Covian Specialty Medical Centre laboratory and handled according to the local standards of practice. HbA1c and high-sensitivity C-reactive protein (hs-CRP) were determined using a selective modular analyser (Roche Corporation). Fibrinogen was measured by nephelometry (DADEBehring Corporation). Accreditation National Entity (ENAC) validated the quality control of the laboratory (accreditation number: 742/LE1586).

#### 2.1.4. Components of Physical Fitness Assessments (Balance, Flexibility, Strength, Endurance) 

Dynamic Balance—tiptoe along a straight line. Subjects tiptoed along one line measuring 10 m and the number of errors (foot outside line) were registered [24]. Reference values are not available.

Static Balance—the one-leg stance duration, with a maximum of 60 s, was measured. It has been established that a recorded duration of less than 5 s implies a great risk of falls [25]. For the analysis of this variable, the mean value between both legs was calculated.

Flexibility—finger floor distance. Subjects were standing and bent forward. The examiner measured the distance from the floor to the fingertips. A finger-floor distance of −1 cm has been previously registered in healthy middle age women [26].

Abdominal strength resistance—trunk flexor (abdominal) muscular strength resistance was assessed by the one-minute curl-up test. The test was conducted as follows: arms were placed at the sides, palms facing down. Feet were flat on the ground with knees bent at 90°. Subjects did curl-ups to lift the shoulder blades until fingertips touched the knees. Subjects performed as many curl-ups as possible in one minute. Previously, 18 repetitions/minute have been registered in healthy middle-aged women [27].

Strength resistance of lower limbs—lower limb muscular strength resistance was assessed by the one-minute squats test. The test was conducted as follows: subjects were standing in front of a chair, facing away from it, with feet shoulders width apart. They squatted down and touch the chair with their backside before standing back up. Number of squats completed in one minute was recorded. A range of 29–32 repetitions/minute have been previously registered in healthy middle age women [28].

Strength resistance of upper limbs—upper limb muscular strength resistance was assessed by the one-minute standing push-ups test [29]. The test was conducted as follows: subjects stood facing a wall, at a similar distance to upper limbs length. They placed hands at shoulder height, arms straight and shoulder width apart, on the wall. They lowered the body until the elbows reached 90°, and then they returned to the starting position with the arms fully extended. Push-ups performed in one minute were counted. Reference values are not available.

Cardiovascular endurance: cardiovascular endurance was measured using a 12-minute endurance walking test or a 12 min Cooper test [30]. Subjects walked as far as they could in 12 min on a 100 m circular path. Number of laps was recorded. Previously, 9.5 laps has been registered in women between 50–60 years [31].

Clinical history, amount of daily physical activity, anthropometry and components of physical fitness (balance, flexibility, strength, endurance) were registered in the same session. Laboratory assessments were taken a mean of 3.7 days before or after this evaluation session.

### 2.2. Statistical Analysis 

Analyses were performed with SPSS software package version 22.0 (SPSS Inc. Chicago, IL, USA). The statistical study was performed following the principles of intention-to-treat analysis, without attributing values to missing data. Mean, standard deviation (SD) and maximum and minimum values were calculated for variables across categories (physically active/sedentary subjects) and statistical significance of the differences was assessed with the Student’s test if the data were normally distributed and with Mann–Whitney-U test when data were not normally distributed. Data distribution was analysed with the Shapiro–Wilk test. Statistical significance was set at two-sided *p* < 0.05. 

We performed regression modelling with General Linear Model Univariate analysis procedure from SPSS. The General Linear Model Univariate procedure allows modelling of the value of a dependent scale variable based on its relationship with categorical and scale variables. We modelled the value of the following dependent variables: BMI, waist circumference, HbA1c, fibrinogen and hs-CRP. The independent variable was physical activity (physically active or sedentary). Adjustment variables were age and each of the physical fitness components: dynamic balance, static balance, flexibility, abdominal strength resistance, strength resistance of lower limbs, resistance strength of upper limbs and cardiovascular endurance. Statistical significance was set at two-sided *p* < 0.05. 

The power and effect size for the inferential tests (Student’s *t* test, Mann–Whitney-U and General Linear Model Univariate analysis) were calculated in a post hoc way.

Graphical representation of significant effects on the General Linear Models were created with observed values in scatterplots including trend lines using Excel software (Microsoft, Redmond, WA, USA). 

## 3. Results

A total of 28 subjects without diagnosis of diabetic polyneuropathy and with a mean age of 58.5 ± 7.8 were recruited. Overall, 16 subjects were physically active and 12 were sedentary. Descriptions of anthropometrics, metabolic profiles and physical fitness measurements, reference values when available, and the percentage of the sample in these values are included in Table 1. Comparisons taking being active or sedentary as the independent variable produced a statistically significant result in the variables BMI and cardiovascular endurance (Table 1). 

None of the General Linear Models showed statistical significance for age or physical activity (Table 2). 

The models that tested BMI and waist circumference as dependent variables did not show any statistical significance for the adjustment variables of physical fitness components.

The models that tested HbA1c, fibrinogen and hs-CRP as dependent variables showed statistical significance for the adjustment variable dynamic balance (Table 2).

**Figure 1:**
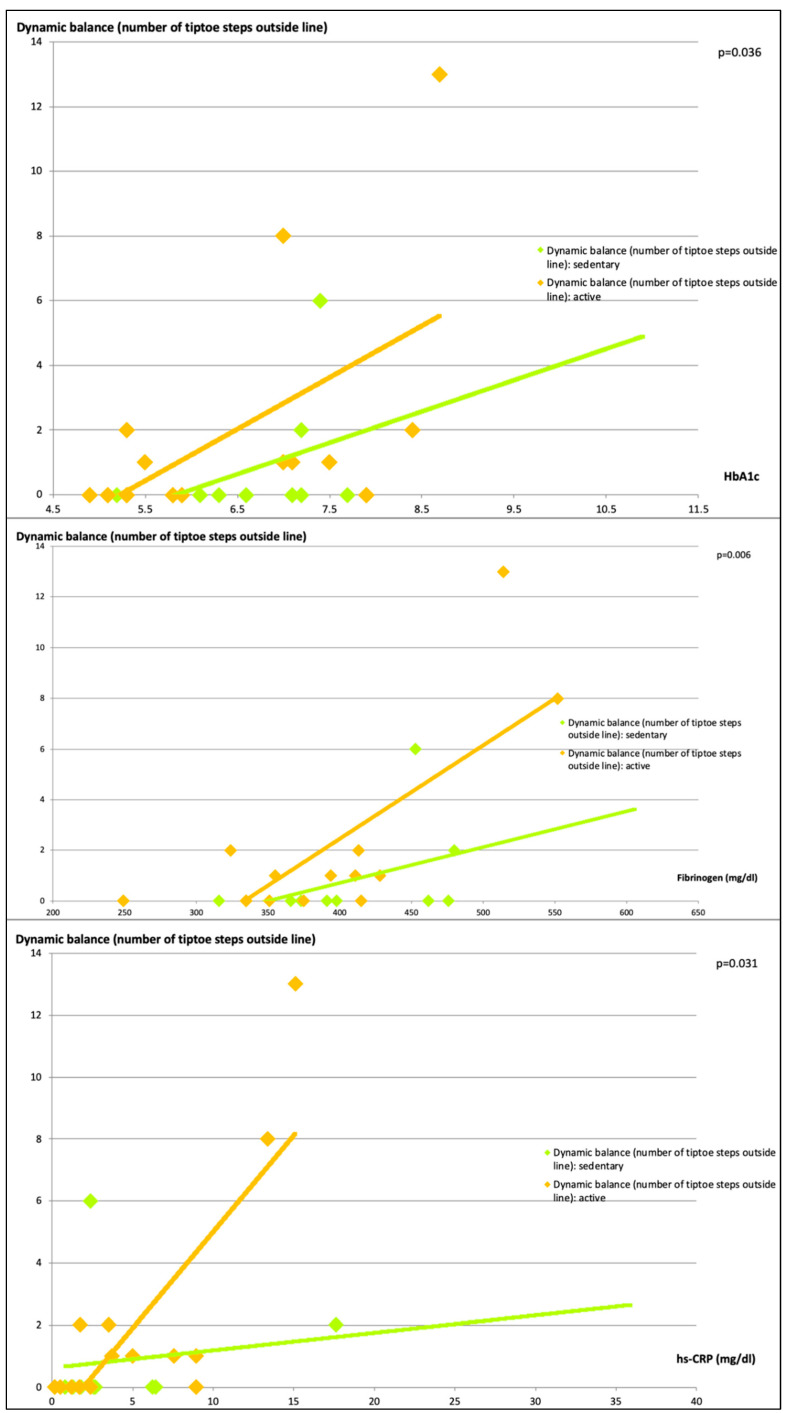
Impaired dynamic balance in subjects with higher HbA1c, fibrinogen and hs-CRP.

Impaired dynamic balance was observed in subjects with higher HbA1c, fibrinogen and hs-CRP (Figure 1).

## 4. Discussion

Active subjects (57.1%) had significantly lower BMI and better cardiovascular endurance than sedentary subjects. The comparisons of the hs-CRP, dynamic and static balance and flexibility variables between active and sedentary subjects showed low power. Thus, the results of the comparisons may have been different in a larger sample. General Linear Models presented high values for power and effect size. They did not show an influence of age or physical activity amount, but HbA1c, fibrinogen and hs-CRP were related with worse dynamic balance.

Obesity has typically been considered as a risk factor for type 2 diabetes. However, it has been suggested that BMI could behave more as a precipitating factor than a trigger for the progression to type 2 diabetes mellitus [32]. The World Health Organization defines overweight as a BMI of 25–29.9 and obesity as a BMI equal to or greater than 30 [33]. Sedentary subjects had grade I obesity (BMI mean: 38.9) [34], and physically active members had grade II overweight (BMI mean: 34.2) (*p* = 0.033) [34]. Both groups, sedentary and active, showed mean values of BMI higher than those previously recorded in similar samples [35,36]. Moderate physical activity performed by the subjects had some effectiveness in controlling excess weight, which supports the existing evidence on the effectiveness of exercise in this regard [37]. A recent review has shown that replacing sedentary time with moderate to vigorous physical activity may be beneficial for reductions in BMI [38].

In total, 85.2% of the sample was within reference values for cardiovascular endurance. Active subjects showed a greater performance in cardiovascular endurance tests. These results advise the inclusion of aerobic training within the exercise routine in diabetics [39].

Waist circumference was above reference values in 94.7% of the sample, therefore central body fat distribution increased waist circumference. Waist circumference in active women in this study was similar to that found in a previous study (104.02 ± 2.05) with a similar sample [36], but sedentary subjects of this study had higher waist circumference. This fat distribution is often linked to an increase in the risk of cardiovascular disease, dyslipidaemia and insulin resistance [11].

Overall, 53.8% of the sample complied with ADA recommendations for HbA1c values (HbA1c aim value is <7%) [40]. HbA1c values in our sample were better than those registered in a previous study with a similar sample (9%) [41]. Higher HbA1c was associated in our sample with worse tiptoe dynamic balance. Diabetic neuropathy is one of the most common complications of diabetes mellitus, distal sensory predominant polyneuropathy being the most common. Symptoms can progress slowly, and they include decreased sensibility [42]. Decreased sensibility, so incipient that it might be asymptomatic in daily life activities, may explain this impaired dynamic balance observed in women of our study. Implementing balance exercises to the training routine of the sample may be clinically relevant for the prevention of falls [43].

Our sample had slightly elevated fibrinogen values (407.9 mg/dL). It has been shown that fibrinogen is an independent risk factor for cardiovascular diseases and appears to be associated with traditional cardiovascular risk factors. Fibrinogen behaves as a major cardiovascular risk factor, similar to hypertension and diabetes. In some studies, it seems to be more of a cardiovascular factor in men than in women, but it is important in both sexes regarding the risk of ischemic heart disease [44]. The importance of fibrinogen with respect to ischemic heart disease was shown in the Strong Heart Study, in which diabetic women, with higher values of fibrinogen than men, showed higher prevalence of myocardial infarction and cardiovascular disease than men [45]. In the diabetic population, the increase in cardiovascular risk seems to be related to the coexistence of several risk factors [46] including haemostasis disturbances with increased fibrinogen, factors VII and VIII and abnormalities in platelet function. Fibrinogen could be the cellular mediator through which cardiovascular risk factors exert their effects [47].

Higher fibrinogen was associated in our sample with worse tiptoe dynamic balance. Additionally, in this case, decreased sensibility might be behind the impaired dynamic balance. Although the precise aetiology of diabetes neuropathy remains unknown, the hypotheses state that diabetes affects the peripheral nerves by several mechanisms. One mechanism is that microangiopathy and arteriosclerosis affecting the vasa nervorum cause ischemia to neurons and axons [42]. Stimulation of atherogenesis may be due to modifications of lipids, complement activation (enhancing the inflammatory response), endothelial dysfunction and hypercoagulation (elevated fibrinogen and plasminogen activator inhibitor) [48].

The hs-CRP marker of inflammation, vascular complications and ischemic heart disease [49] was found to be above normal values (5 mg/dL) in this sample, with a mean of 6.1 mg/dL. hs-CRP values were higher in our sample than in previous studies [50]. Higher hs-CRP was associated in our sample with worse tiptoe dynamic balance. Again, decreased sensibility might be behind impaired dynamic balance. Inflammation, oxidative stress, and mitochondrial dysfunction have been linked to the aetiology of diabetic polyneuropathy [51]. The inflammatory process induces oxidative stress and reduces cellular antioxidant capacity [52]. Oxidative stress, with excessive accumulation of glycoproteins and polyol flux, induces axonal degeneration [42]. Although the mechanism of the relationship between CRP and hyperglycaemia is not completely clear, it has been shown that higher levels of CRP are associated with diabetic polyneuropathy [53]. Subclinical diabetic polyneuropathy in diabetic women can alter balance function, so balance training may optimize the health care of these patients. 

This study has several limitations. It is limited by a relatively small sample size and by the cross-sectional design conducted in a single medical centre. Associations have been examined, but no causal relationship can be estimated. Future larger cohort and prospective studies might support our findings. However, the current findings may be relevant for the development of exercise recommendations for the diabetic population.

## 5. Conclusions

Active women had lower BMI and showed a better performance in cardiovascular endurance. No differences were observed between active and sedentary women in age, waist circumference, HbA1c, fibrinogen, hs-CRP, dynamic balance, static balance, flexibility or abdominal, lower limb and upper limb strength resistance. Tiptoe dynamic balance impairments were related to worse glycaemic control, hypercoagulation and inflammatory state.

## Figures and Tables

**Table 1:** Descriptive study and comparative analysis between physically active and sedentary subjects.

	All Subjects Mean ± SD (Min–Max)	Reference Values	Percentage Values of the Sample within Reference	Physically Active Subjects Mean ± SD (Min–Max)	Sedentary Subjects Mean ± SD (Min–Max)	*p* Value	Effect Size d	Power
Age (years)	58.5 ± 7.8 (46.0–76.0)			57.7 ± 8.1 (46.0–76.0)	59.4 ± 7.7 (47.0–76.0)	0.586	0.218	0.287
BMI	36.2 ± 5.9 (24.9–48.8)	18.5–24.9 (Salas-Salvado. 2007)	3.6%	34.2 ± 5.7 (24.9–43.6)	38.9 ± 5.1 (29.5–48.8)	0.033	0.797	0.989
Waist circumference (cm)	107 ± 12.6 (76.0–128.3)	<88 (Third Report of the National Cholesterol Education Program. 2002)	7.1%	103.1 ± 12.6 (76.0–120.8)	112.3 ± 11.0 (88.6–128.3)	0.055	0.73	1.66
HbA1c (%)	6.8 ± 1.4 (4.9–10.9)	<5.7% ref./7% target (ADA. 2012)	26.9%/53.8%	6.5 ± 1.3 (4.9–8.7)	7.2 ± 1.6 (5.2–10.9)	0.243	0.5	0.799
Fibrinogen (mg/dL)	407.9 ± 77.1 (249.0–606.0)	200–400 (Gailani D. 2008)	50%	389.4 ± 77.4 (249.0–552.0)	429.6 ± 74.1 (316.0–606.0)	0.191	0.521	0.827
hs-CRP (mg/dL)	6.1 ± 7.6 (0.2–35.9)	<5 (Hayashino. 2014)	65.4%	5.3 ± 4.8 (0.2–15.1)	7.0 ± 10.2 (0.8–35.9)	0.877	0.224	0.288
Dynamic balance: line test (number of tiptoe steps outside line)	1.7 ± 3.2 (0.0–13.0)	Not available		2.1 ± 3.6 (0.0–13.0)	0.9± 2.0 (0.0–6.0)	0.179	0.375	0.569
Static balance: unipodal (s)	25.5± 18.9 (0.0–60.0)	<5 s high risk of falls (Menendez. 2005)	80.8%	26.3 ± 18.5 (1.0–60.0)	24.1 ± 20.5 (0.0–60.0)	0.786	0.116	0.143
Flexibility: trunk anterior flexion (cm)	10.9 ± 8 (−10.0–25.0)	(−) 1 cm (Zaproudina.2006)	7.4%	11.2 ± 9.2 (−10.0–25.0)	10.3 ± 6.3 (−2.5–18.0)	0.796	0.113	0.139
Abdominal strength resistance (repetitions/minute)	5.8 ± 10.4 (0.0–32.0)	18 (Sekendiz. 2010)	22.2%	8.3 ± 11.6 (0.0–32.0)	2.2 ± 7.2 (0.0–24.0)	0.124	0.587	0.885
Strength resistance of lower limbs (repetitions/minute)	18.9 ± 5.6 (0.0–31.0)	29–32 (Mackenzie. 2005)	3.7%	20.7 ± 3.8 (17.0–31.0)	16.1 ± 6.7 (0.0–22.0)	0.13	0.839	0.992
Strength resistance of upper limbs (repetitions/minute)	27.0 ± 6.7 (12.0–39.0)	Not available		28.5 ± 7.1 (17.0–39.0)	24.7 ± 5.8 (12.0–33.0)	0.157	0.567	0.879
Cardiovascular endurance (number of 100 m laps in 12 min)	12.2 ± 2.9 (3.5–17.5)	9.5 (Salbach. 2015)	85.2%	13.2 ± 2.3 (8.5–17.5)	10.7 ± 3.2 (3.5–14.5)	0.025	0.862	0.996

**Table 2:** General Linear Models parameter estimates.

	Unstandardized Coefficients				95% Confidence Interval	
	Estimate	Standard Error	*t*-Statistic	*p* Value	Lower Bound	Upper Bound
**Dependent Variable: BMI**						
Effects Size f: 0.910 Power: 0.988 *n* = 25						
Constant	59.712	17.980	3.321	0.005	21.389	98.035
Dynamic balance: line test (number of tiptoe steps outside line)	−0.015	0.434	−0.034	0.973	−0.940	0.910
Static balance: unipodal (s)	−0.177	0.106	−1.670	0.116	−0.403	0.049
Flexibility: trunk anterior flexion (cm)	−0.056	0.170	−0.329	0.747	−0.418	0.306
Abdominal strength resistance (repetitions/minute)	−0.208	0.139	−1.492	0.156	−0.504	0.089
Strength resistance of lower limbs (repetitions/minute)	0.201	0.287	0.701	0.494	−0.410	0.812
Strength resistance of upper limbs (repetitions/minute)	0.007	0.204	0.034	0.973	−0.427	0.441
Cardiovascular endurance (number of 100 m laps in 12 min)	−0.076	0.673	−0.112	0.912	−1.511	1.360
Age	−0.378	0.201	−1.886	0.079	−0.806	0.049
Physical Activity	2.977	2.970	1.002	0.332	−3.353	9.308
**Dependent Variable: Waist circumference (cm)**						
Effects size f: 1.037 Power: 0.998 *n* = 25						
Constant	126.200	37.577	3.358	0.004	46.106	206.293
Dynamic balance: line test (number of tiptoe steps outside line)	0.573	0.907	0.631	0.537	−1.361	2.506
Static balance: unipodal (s)	−0.424	0.221	−1.915	0.075	−0.896	0.048
Flexibility: trunk anterior flexion (cm)	−0.057	0.355	−0.162	0.874	−0.813	0.699
Abdominal strength resistance (repetitions/minute)	−0.612	0.291	−2.104	0.053	−1.232	0.008
Strength resistance of lower limbs (repetitions/minute)	0.695	0.599	1.160	0.264	−0.582	1.972
Strength resistance of upper limbs (repetitions/minute)	0.345	0.426	0.810	0.430	−0.563	1.253
Cardiovascular endurance (number of 100 m laps in 12 min)	0.783	1.407	0.557	0.586	−2.216	3.783
Age	−0.727	0.419	−1.734	0.103	−1.621	0.167
Physical Activity	8.969	6.208	1.445	0.169	−4.262	22.200
**Dependent Variable: HbA1c (%)**						
Effects size f: 0.883 Power: 0.974 *n* = 23						
Constant	4.096	5.047	0.812	0.432	−6.807	14.998
Dynamic balance: line test (number of tiptoe steps outside line)	0.222	0.095	2.336	0.036	0.017	0.427
Static balance: unipodal (s)	0.007	0.023	0.310	0.761	−0.043	0.057
Flexibility: trunk anterior flexion (cm)	0.030	0.046	0.661	0.520	−0.069	0.129
Abdominal strength resistance (repetitions/minute)	−0.016	0.032	−0.494	0.629	−0.085	0.053
Strength resistance of lower limbs (repetitions/minute)	−0.037	0.063	−0.581	0.571	−0.174	0.100
Strength resistance of upper limbs (repetitions/minute)	0.057	0.046	1.220	0.244	−0.044	0.157
Cardiovascular endurance (number of 100 m laps in 12 min)	0.038	0.144	0.266	0.794	−0.273	0.349
Age	0.004	0.059	0.061	0.952	−0.123	0.130
Physical Activity	0.432	0.654	0.662	0.520	−0.979	1.844
**Dependent Variable: Fibrinogen (mg/dL)**						
Effects size f: 1.431 Power: 0.999 *n* = 23						
Constant	250.022	244.670	1.022	0.325	−278.554	778.599
Dynamic balance: line test (number of tiptoe steps outside line)	15.216	4.601	3.307	0.006	5.277	25.156
Static balance: unipodal (s)	−0.022	1.127	−0.019	0.985	−2.456	2.412
Flexibility: trunk anterior flexion (cm)	2.089	2.221	0.940	0.364	−2.709	6.887
Abdominal strength resistance (repetitions/minute)	1.290	1.556	0.829	0.422	−2.072	4.651
Strength resistance of lower limbs (repetitions/minute)	−2.054	3.077	−0.668	0.516	−8.702	4.594
Strength resistance of upper limbs (repetitions/minute)	4.498	2.247	2.002	0.067	−0.356	9.351
Cardiovascular endurance (number of 100 m laps in 12 min)	−4.456	6.980	−0.638	0.534	−19.535	10.622
Age	0.787	2.843	0.277	0.786	−5.355	6.928
Physical Activity	46.225	31.683	1.459	0.168	−22.221	114.671
**Dependent Variable: hs-CRP (mg/dL)**						
Effects size f: 1.094 Power: 0.998 *n* = 23						
Constant	9.289	20.081	0.463	0.651	−34.094	52.672
Dynamic balance: line test (number of tiptoe steps outside line)	0.912	0.378	2.415	0.031	0.096	1.728
Static balance: unipodal (s)	−0.046	0.092	−0.502	0.624	−0.246	0.153
Flexibility: trunk anterior flexion (cm)	0.131	0.182	0.0718	0.485	−0.263	0.525
Abdominal strength resistance (repetitions/minute)	−0.160	0.128	−1.256	0.231	−0.436	0.115
Strength resistance of lower limbs (repetitions/minute)	0.331	0.253	1.310	0.213	−0.215	0.877
Strength resistance of upper limbs (repetitions/minute)	0.131	0.184	0.713	0.488	−0.267	0.530
Cardiovascular endurance (number of 100 m laps in 12 min)	−0.196	0.573	−0.342	0.738	−1.434	1.042
Age	−0.221	0.233	−0.949	0.360	−0.725	0.283
Physical Activity	0.714	2.600	0.274	0.788	−4.904	6.331

## Data Availability

The dataset generated and analysed during the current study is not publicly available because it includes clinical subject data. Thus, this data may only be publicly shared and presented in aggregate anonymous form. However, the dataset is available from the corresponding author upon reasonable request.
